# Patient and provider factors associated with colorectal cancer screening among average risk health plan enrollees in the US, 2015–2018

**DOI:** 10.1186/s12913-023-09474-9

**Published:** 2023-05-26

**Authors:** Nicole M. Engel-Nitz, Lesley-Ann Miller-Wilson, Lisa Le, Paul Limburg, Deborah A. Fisher

**Affiliations:** 1 Optum, Eden Prairie, MN USA; 2 11000 Optum Circle Eden Prairie, 952-205-7770, Eden Prairie, MN 55344 USA; 3 Exact Sciences, Madison, WI USA; 4 Mayo Clinic, Rochester, MN USA; 5 Duke University, Durham, NC United States

**Keywords:** Screening, Colorectal cancer, Patient characteristics, Provider characteristics

## Abstract

**Background:**

To assess patient and primary care provider (PCP) factors associated with adherence to American Cancer Society (ACS) and United States Preventive Services Task Force (USPSTF) guidelines for average risk colorectal cancer (CRC) screening.

**Methods:**

Retrospective case-control study of medical and pharmacy claims from the Optum Research Database from 01/01/2014 − 12/31/2018. Enrollee sample was adults aged 50 − 75 years with ≥ 24 months continuous health plan enrollment. Provider sample was PCPs listed on the claims of average-risk patients in the enrollee sample. Enrollee-level screening opportunities were based on their exposure to the healthcare system during the baseline year. Screening adherence, calculated at the PCP level, was the percent of average-risk patients up to date with screening recommendations each year. Logistic regression modelling was used to examine the association between receipt of screening and enrollee and PCP characteristics. An ordinary least squares model was used to determine the association between screening adherence among the PCP’s panel of patients and patient characteristics.

**Results:**

Among patients with a PCP, adherence to ACS and USPSTF screening guidelines ranged from 69 to 80% depending on PCP specialty and type. The greatest enrollee-level predictors for CRC screening were having a primary/preventive care visit (OR = 4.47, p < 0.001) and a main PCP (OR = 2.69, p < 0.001).

**Conclusions:**

Increased access to preventive/primary care visits could improve CRC screening rates; however, interventions not dependent on healthcare system contact, such as home-based screening, may circumvent the dependence on primary care visits to complete CRC screening.

**Supplementary Information:**

The online version contains supplementary material available at 10.1186/s12913-023-09474-9.

## Introduction

Approximately 4% of the US population will develop colorectal cancer (CRC) over their lifetime [1]. Early-stage CRC is highly treatable; however, only 37% of people are diagnosed with localized CRC [2]. When diagnosed with localized disease, the a 5-year survival rate is approximately 91%; however, this drops to 15% when distant metastases are present at diagnosis [3].

The American Cancer Society (ACS) and the United States Preventive Services Task Force (USPSTF) recommend CRC screening for average-risk individuals beginning at 45 years of age using stool, imaging, or endoscopy tests. [4, 5] Screening can detect neoplasia at an earlier, more treatable stage and prevent CRC through identification and removal of pre-invasive lesions (e.g., adenomatous polyps); however, 30.3% of eligible US adults are not up to date with CRC screening [6]. In previous studies, fear, lack of provider recommendation, and lack of patient knowledge about screening were cited as the top patient-reported barriers to CRC screening. [7, 8] CRC screening adherence increased when patients were given a choice of screening modality [9].

Several studies have investigated patient- and provider-level characteristics associated with CRC screening adherence. In previous studies, patient characteristics associated with higher CRC screening participation rates included male sex, white race, being married, non-obese, higher education levels, and higher socioeconomic status. [10–14] Provider factors associated with higher adherence to CRC screening guidelines include a larger panel of patients eligible for screening, working in a facility that is a shorter distance to a colonoscopy center, and providing time and reminders for CRC screening discussions with patients [15, 16].

The purpose of this study was to assess and update patient and provider factors associated with adherence to ACS and USPSTF guidelines for average risk CRC screening in the context of currently available screening modalities, including the newer Cologuard test (FDA approved in 2014). Large-scale survey studies, including data on CRC screening rates collected by the Behavior Risk Factor Surveillance System (BRFSS) [17] and National Health Interview Survey (NHIS) [18], rely on patient self-report on whether they’ve received screening and the timing of screening. Previous studies have suggested self-report of CRC screening has a moderate level of validity [19–21] and reliability [22] with a tendency towards over-reporting. The data used in the current study provides a more accurate assessment of the timing of screening tests with confirmed dates of service.

## Methods

### Study design and data source

In this retrospective case control study, medical and pharmacy claims and enrollment information from January 01, 2014 to December 31, 2018 (study period) were obtained from US commercial and Medicare Advantage health plan members in the Optum Research Database (ORD). The ORD is a de-identified, diverse, and nationally representative administrative claims database containing approximately 9% of the US commercially insured population as of 2017. Institutional review board approval or waiver of approval was not required for this study because the study data were secondary and de-identified in accordance with the United States Department of Health and Human Services (HHS Privacy Rule’s requirements for de-identification codified at 45 C.F.R. § 164.514(b)). Two study samples were identified: a population of eligible health plan enrollees (enrollees), and a population of primary care providers. Medical claims for screening tests were identified through International Classification of Diseases, 9th and 10th Revisions, Clinical Modification (ICD-9-CM/ICD-10-CM) procedure codes (ICD-0-PCS/ICD-10-PCS) and Current Procedural Terminology (CPT) codes.

### Enrollee sample selection

The enrollee study sample consisted of adults aged 50 to 75 years during the calendar year of the analysis with continuous enrollment in the health plan with medical and pharmacy benefits for ≥ 12 months (up to 10 years) prior to the year of the analysis (baseline period) through the entire year of the analysis (minimum of 24 months of enrollment [follow-up period]). The most recent, available, complete year of enrollment was used as the year of analysis for each enrollee. Enrollees were excluded if they had evidence of conditions indicating a higher risk for CRC (i.e., ≥ 1 medical claim during the baseline period with a diagnosis code in any position on the claim for adenoma, sessile serrated polyp, prior diagnoses of CRC) or a personal history of or diagnosis of inflammatory bowel disease at any time during the study period; or a family history of CRC/gastrointestinal cancer.

### Enrollee screening status cohorts

The most recent full calendar year of available data was used to assign screening status to the enrollee population. Screening was based on the most recent ACS and USPSTF guidelines using a stool-based test (i.e., fecal immunochemical test [FIT], guaiac-based fecal occult blood test [gFOBT], multi-targeted stool DNA test [mt-sDNA]) or visual exams of the colon and rectum (i.e., colonoscopy, CT colonography, flexible sigmoidoscopy) [4]. Enrollees in the screened cohort had ≥ 1 CPT/ICD-9-CM/ICD-10-CM procedural code during the analysis year for colonoscopy, mt-sDNA, FIT, gFOBT, CT colonography or flexible sigmoidoscopy, or barium enema. The index year was the last available full calendar year with evidence of screening for the enrollee. Enrollees in the unscreened cohort were a control population of randomly selected enrollees who did not have high-risk conditions for CRC and did not have a claim for a CRC screening test during the analysis year or any previous study year. The index date for enrollees in the unscreened cohort was selected to mirror the month/year distribution of index dates among enrollees in the screened cohort. Enrollees who did not have CRC screening in the year of analysis but had screening in previous years were not included in the analysis.

### Provider sample

The provider sample included primary care providers (i.e., family/general practice, internal medicine, obstetrics/gynecology [OB/GYN], geriatrician, nurse practitioner, physician assistant) who were listed on the claims of average-risk patients (i.e., no evidence of high-risk CRC conditions) in the enrollee sample between January 01, 2014 through December 31, 2018. Nurse practitioners and physician assistants were included if they were listed on ≥ 1 claim for a preventive health care service. Each provider was required to have seen ≥ 10 patients from the enrollee sample to ensure the sample was representative of the provider’s broader patient caseload of insured patients and to provide a sufficient patient sample for calculating adherence rates unique to each provider.

### Study measures

#### Enrollee-level measures

Enrollee demographic and clinical characteristics were measured during the baseline period and included age, gender, insurance type, geographic region, urbanicity, race/ethnicity, education level, income, net worth, health plan type, indication of consumer-driven healthcare (i.e., health reimbursement arrangement, health savings account), Charlson comorbidity score, and Agency for Healthcare Research and Quality comorbidities.

Screening opportunities were measured based on the enrollee’s exposure to the health care system during the baseline year and included having at least one primary or preventive care visit, the presence of a main primary care provider (visits to only one provider or, if multiple providers, visited one provider more than others), and the presence of claims for preventive care visits.

#### Provider-level measures

Provider characteristics measured during the baseline period included provider specialty, gender, ethnicity, total number of patients in the ORD, and mean number of visit days per patient. Patient characteristics by provider were also measured and included the count of patients with any claim for CRC and the percent of patients by ethnicity category, income and net worth level, rural versus urban living, gender/age category/mean age, geographic region, and influenza vaccination status during the baseline calendar year. The count of patients with a CRC diagnosis was a measure of provider exposure to CRC to determine whether providers who were exposed to patients with CRC would be more adherent to CRC screening guidelines. Influenza vaccination was used as an indicator of general preventive care.

Screening adherence was calculated at the provider level as the percent of average-risk patients who were up to date with screening recommendations each calendar year. A patient was considered up to date if they had evidence of a colonoscopy within 10 years, CT colonography or flexible sigmoidoscopy within 5 years, mt-sDNA within 3 years, or FIT/gFOBT within 1 year.

### Analysis

#### Enrollee-level analysis

Results were stratified by screening status. Bivariate comparisons of baseline characteristics and outcomes measures were calculated using the appropriate test based on the distribution of the measure (e.g., odds ratio [OR], t-test, f-test, Wilcoxon rank-sum, chi-square test).

A logistic regression model was developed to determine the association between receipt of screening and enrollee demographic and clinical characteristics, enrollee socioeconomic status (SES), and provider characteristics. An additional model was also estimated including only women to assess the impact of provider type, specifically OB/GYN, on adherence rates.

#### Provider-level analysis

In the provider analyses, the cumulative incidence was calculated for each provider to assess the proportion of eligible patients who received CRC screening for each provider, and the characteristics of the provider and their patient panel (as observed in the claims data) were assessed. Patients who saw multiple providers of different types in the selected provider population counted towards each providers’ numbers.

An ordinary least squares (OLS) model was estimated to determine the association between screening adherence among the physician’s panel of patients and physician demographics, patient demographic and clinical characteristics, and patient SES.

## Results

### Enrollee-level results

#### Baseline demographic and clinical characteristics

**Fig. 1:**
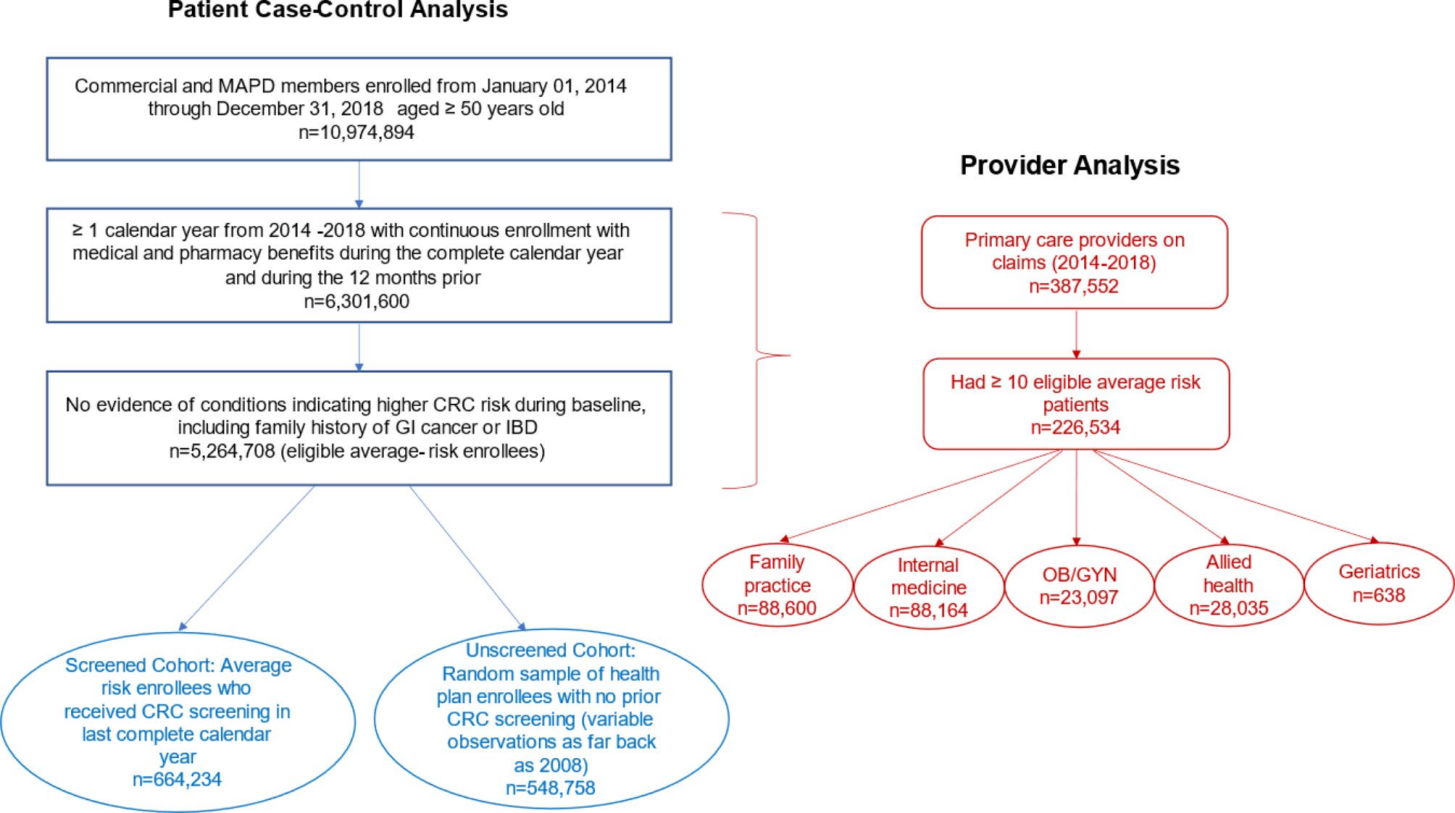
Health plan enrollee and provider sample selection

**Fig. 2:**
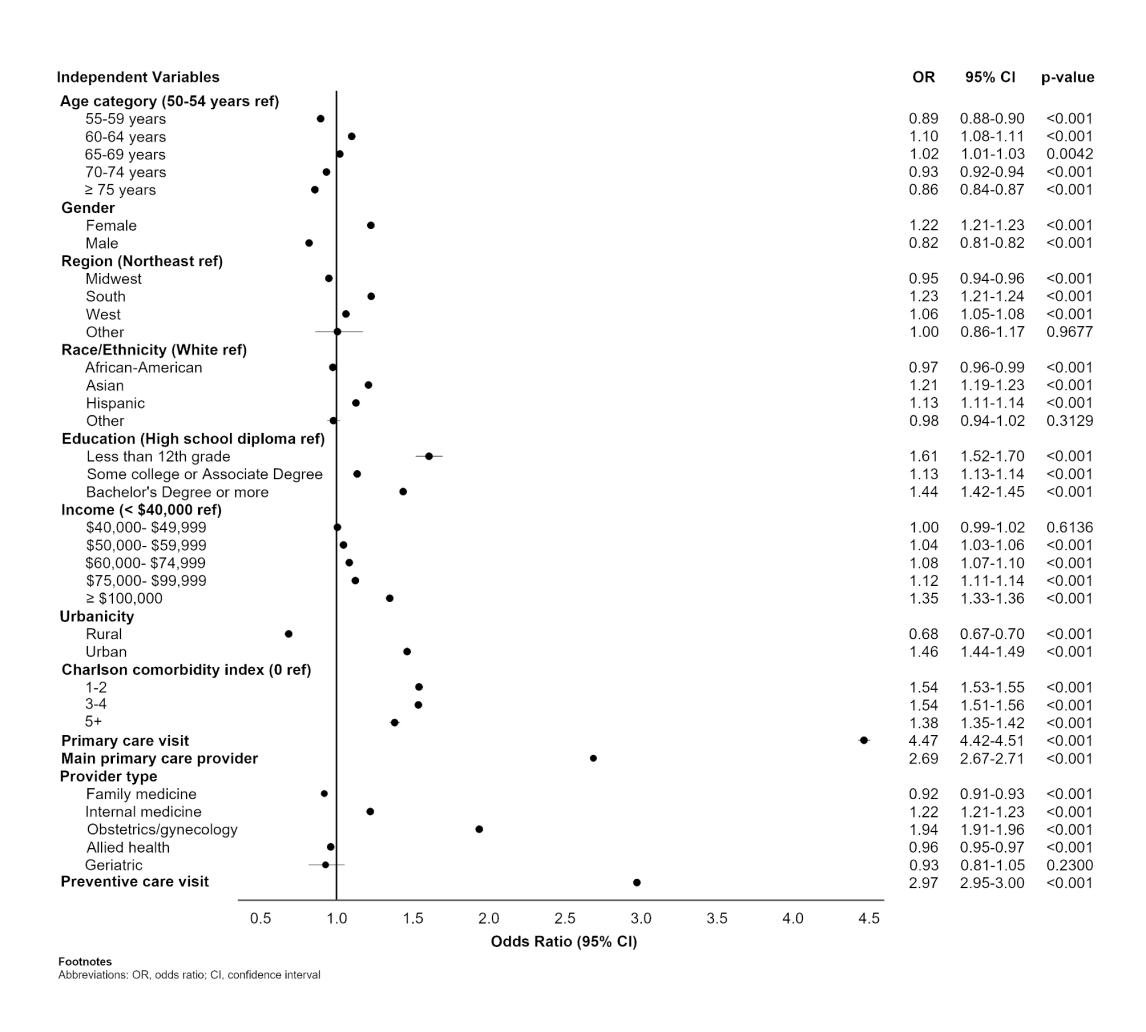
Unadjusted health plan enrollee demographic and clinical characteristics

A total of 664,234 screened and 548,758 unscreened enrollees were included in the enrollee analysis (Fig. 1). Baseline enrollee demographics and clinical characteristics are shown in Fig. 2.

#### CRC screening and odds of healthcare utilization

Enrollees who were screened had greater odds of having a primary or preventive care visit and having a main primary care provider (Fig. 2). The odds of having ≥ 1 primary care visit or preventive care visit was 4.47 (95% CI = 4.42‒4.51, p < 0.001) and 2.97 (95% CI = 2.95‒3.00, p < 0.001) times greater, respectively, among screened versus unscreened enrollees. Additionally, screened enrollees had 2.69 times greater odds of having a main primary care provider than unscreened enrollees (95% CI = 2.67‒2.71, p < 0.001). Visits to an internal medicine provider or an OB/GYN were associated with 1.22 (95% CI = 1.21‒1.23, p < 0.001) and 1.94 (95% CI = 1.91‒1.96, p < 0.001) greater odds of being screened, respectively. Conversely, visits to a family practice or advanced practice provider were associated with lower odds of being screened (p < 0.001 for both comparisons).

**Fig. 3:**
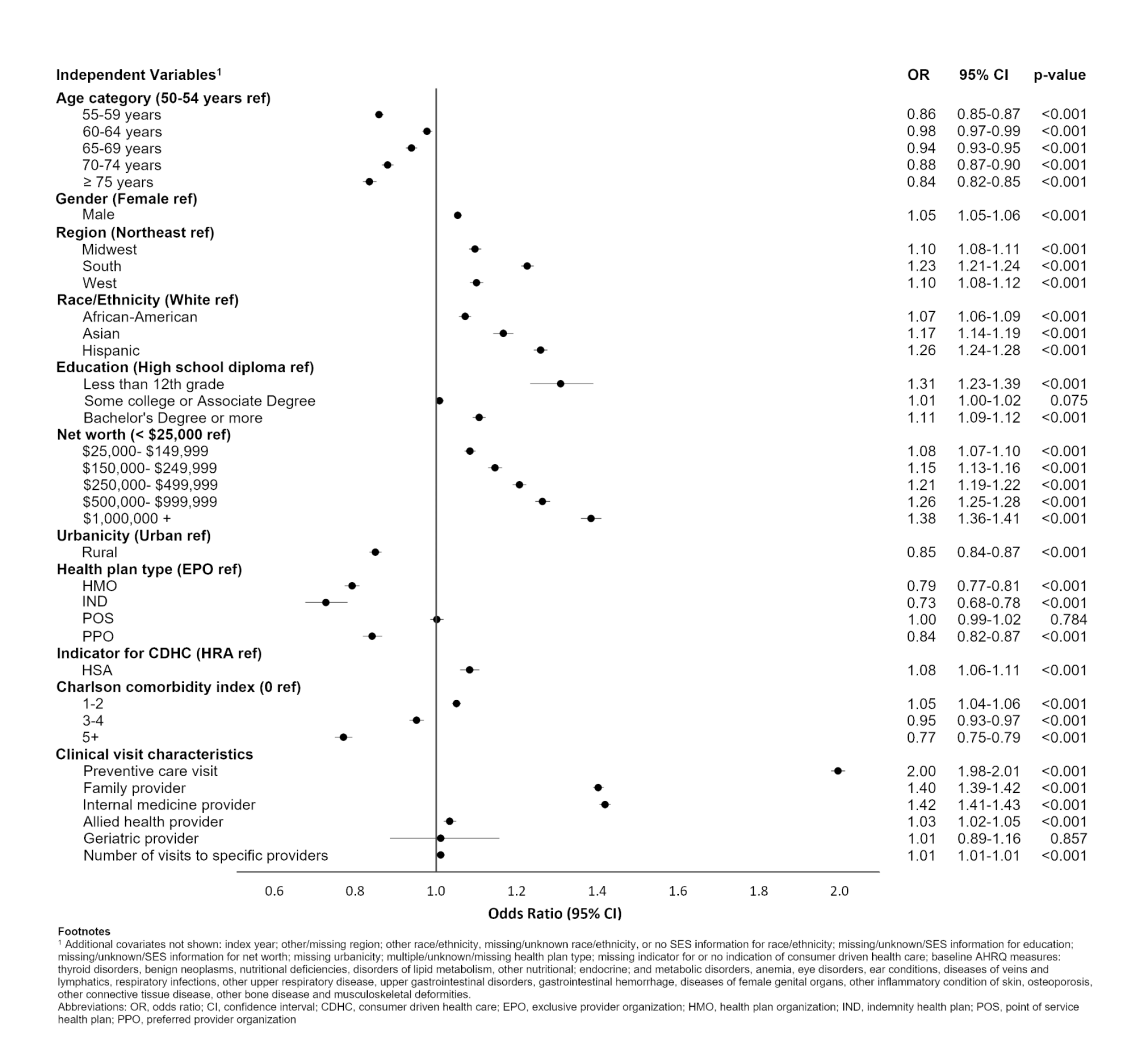
Logistic regression model of the association between health plan enrollee characteristics and CRC screening status

Multivariate model results were consistent, showing the same general effects found in the descriptive analyses (Fig. 3). Of note, male enrollees were slightly more likely than female enrollees to be screened after adjusting for other characteristics. Having visited a primary care provider or having a preventive care visit in the baseline year were positively associated with screening. The effect of the primary care provider varied across provider type, with higher odds of CRC screening among enrollees who were patients of OB/GYNs and internal medicine providers (Fig. 3 and Supplementary Table 1).

### Provider-level results

#### Provider demographic characteristics

Most providers were family practice (39.1%) or internal medicine (38.0%) and had a mean age of 54.4 years (Table 1). Gender varied by specialty with more female OB/GYNs (57.9%) and advanced practice providers (77.1%) than males. Most providers were in an individual practice, which ranged from 98.3% of advanced practice providers to 90.6% of geriatricians.

**Table 1:** Provider Demographics

Demographics	Overall Primary (n = 226,534)	Family (n = 88,600)	Internal Medicine (n = 86,164)	OB/Gyn (n = 23,097)	Advanced Practice (n = 28,035)	Geriatrician (n = 638)
**Age, mean (SD)**	54.4 (11.2)	55.2 (11.3)	54.4 (11.1)	56.1 (10.9)	49.1 (10.4)	54.1 (9.7)
**Male gender, n (%)**	111,360 (49.2)	45,524 (51.4)	50,342 (58.4)	9,230 (40.0)	5,945 (21.2)	319 (50.0)
**Region, n (%)**						
Northeast^1^	40,021 (17.7)	11,671 (13.2)	20,802 (24.1)	4,437 (19.2)	2,968 (10.6)	143 (22.4)
Midwest^2^	61,636 (27.2)	26,121 (29.5)	20,195 (23.4)	5,429 (23.5)	9,738 (34.7)	153 (24.0)
South^3^	87,052 (38.4)	34,526 (39.0)	31,570 (36.6)	9,786 (42.4)	10,910 (38.9)	260 (40.8)
West^4^	37,825 (16.7)	16,282 (18.4)	13,597 (15.8)	3,445 (14.9)	4,419 (15.8)	82 (12.9)
**Race/ethnicity, n (%)**						
White	21,304 (9.4)	8,906 (10.1)	7,181 (8.3)	3,320 (14.4)	1,867 (6.7)	30 (4.7)
African American	494 (0.2)	160 (0.2)	234 (0.3)	73 (0.3)	26 (0.1)	1 (0.2)
Asian	3,590 (1.6)	933 (1.1)	2,252 (2.6)	332 (1.4)	51 (0.2)	22 (3.5)
Hispanic	1,728 (0.8)	651 (0.7)	727 (0.8)	254 (1.1)	88 (0.3)	8 (1.3)
Other	1,033 (0.5)	296 (0.3)	617 (0.7)	91 (0.4)	26 (0.1)	3 (0.5)
Missing	198,385 (87.6)	77,654 (87.7)	75,153 (87.2)	19,027 (82.4)	25,977 (92.7)	574 (90.0)
**Practice type, n (%)**						
Individual	217,700 (96.1)	83,639 (94.4)	83,355 (96.7)	22,568 (97.7)	27,560 (98.3)	578 (90.6)
Hospital	81 (0.04)	38 (0.04)	38 (0.04)	3 (0.01)	2 (0.01)	0 (0.0)
Group practice	7,730 (3.4)	4,266 (4.8)	2,499 (2.9)	488 (2.1)	419 (1.5)	58 (9.1)
Other facility	786 (0.4)	563 (0.6)	173 (0.2)	17 (0.1)	33 (0.1)	0 (0.0)
Unknown/missing	237 (0.1)	94 (0.1)	99 (0.1)	21 (0.1)	21 (0.1)	2 (0.3)

^1^Connecticut, Massachusetts, Maine, New Hampshire, Rhode Island, Vermont, New Jersey, New York, Pennsylvania

^2^Illinois, Indiana, Michigan, Ohio, Wisconsin, Iowa, Kansas, Minnesota, Missouri, North Dakota, Nebraska, South Dakota

^3^Washington DC, Delaware, Florida, Georgia, Maryland, North Carolina, South Carolina, Virginia, West Virginia, Alabama, Kentucky, Mississippi, Tennessee, Arkansas, Louisiana, Oklahoma, Texas

^4^Arizona, Colorado, Idaho, Montana, New Mexico, Nevada, Utah, Wyoming, Alaska, California, Hawaii, Oregon, Washington

#### Patient demographic characteristics by provider type

Demographic and clinical characteristics of the PCP’s patient panels are shown in Table 2. Primary care providers saw a mean of 56.7 average-risk patients and 23.4 high-risk patients during the calendar year (data not shown). Family medicine providers had greater mean average-risk patient counts (62.5) than other providers (p < 0.001 for all comparisons) and internal medicine providers had greater high-risk patient counts (26.0) compared to other providers.

**Table 2:** Demographic characteristics of patient panels by primary care provider type^1^

Demographics		Overall Primary (n = 226,534)	Family (n = 88,600)	Internal Medicine (n = 86,164)	OB/Gyn (n = 23,097)	Advanced Practice (n = 28,035)	Geriatrician (n = 638)
**Number of patients in each provider’s panel** ^2^		252,545	96,784	95,970	25,921	33,159	711
**Age**		63.0 (4.1)	62.4 (3.9)	64.2 (3.8)	59.9 (3.7)	63.6 (4.1)	66.5 (3.9)
**Male gender**		39.2 (18.5)	42.7 (13.7)	45.0 (13.8)	2.1 (8.5)	40.7 (15.8)	41.1 (12.0)
**Region**							
Northeast^3^		17.1 (36.6)	12.7 (32.2)	23.5 (41.1)	18.7 (38.1)	10.3 (29.7)	21.3 (39.9)
Midwest^4^		27.5 (43.4)	29.8 (44.5)	23.8 (41.1)	23.6 (41.5)	35.2 (46.5)	25.3 (42.1)
South^5^		39.0 (47.1)	39.5 (47.2)	37.5 (46.4)	42.8 (48.0)	39.3 (47.3)	40.7 (47.2)
West^6^		16.3 (35.8)	18.0 (37.4)	15.2 (34.7)	14.9 (34.8)	15.2 (34.8)	12.6 (32.2)
**Race/ethnicity**							
White		69.6 (22.4)	71.1 (22.7)	66.6 (22.4)	69.7 (21.8)	73.9 (20.3)	62.0 (22.5)
African American		11.2 (16.6)	10.1 (16.4)	13.1 (17.5)	10.4 (15.2)	9.9 (15.5)	15.7 (19.5)
Asian		2.8 (7.4)	2.4 (6.6)	3.4 (8.7)	3.3 (8.0)	1.4 (3.8)	3.8 (9.1)
Hispanic		8.6 (14.5)	8.7 (15.5)	8.9 (13.9)	9.0 (14.5)	6.7 (12.7)	10.2 (14.8)
Other		0.7 (2.1)	0.7 (2.2)	0.8 (2.1)	0.7 (1.9)	0.6 (1.9)	0.8 (2.1)
Missing		7.1 (7.8)	7.0 (7.9)	7.3 (7.5)	6.9 (8.1)	7.4 (7.9)	7.6 (7.6)
**Education**							
<12 grade		0.4 (2.3)	0.4 (2.6)	0.4 (2.1)	0.3 (1.9)	0.3 (2.1)	0.4 (1.6)
High school diploma		29.5 (23.7)	31.2 (25.5)	28.9 (22.0)	23.1 (21.0)	31.2 (23.8)	30.9 (20.3)
Some college/Associate degree		50.4 (21.4)	51.4 (23.0)	49.1 (19.9)	50.4 (20.0)	51.8 (21.5)	48.8 (18.3)
Bachelor/graduate degree		15.3 (19.2)	12.8 (17.9)	17.1 (20.0)	22.3 (22.3)	12.0 (15.6)	15.2 (17.6)
Education missing		4.4 (7.2)	4.3 (7.4)	4.5 (7.0)	4.0 (7.6)	4.8 (7.3)	4.7 (6.8)
**Net worth**							
<$25,000		24.6 (17.2)	25.3 (17.3)	24.9 (17.2)	18.7 (15.4)	26.0 (17.2)	27.3 (17.0)
$25,000–$149,999		20.4 (11.1)	21.9 (11.4)	18.7 (10.3)	19.2 (11.4)	21.7 (11.1)	18.5 (9.5)
$150,000–$249,999		10.2 (6.8)	10.6 (7.0)	9.6 (6.5)	10.3 (7.0)	10.6 (7.2)	9.6 (6.7)
$250,000–$499,999		14.6 (9.3)	14.5 (9.4)	14.3 (9.0)	16.2 (9.2)	14.5 (9.6)	13.8 (9.2)
$500,000–$999,999		13.0 (11.3)	12.1 (11.4)	13.4 (11.1)	16.8 (11.9)	11.6 (10.5)	11.7 (10.4)
≥$1,000,000		7.9 (12.0)	6.3 (10.6)	9.3 (13.2)	11.6 (14.3)	5.8 (8.9)	6.7 (10.1)
Net worth missing		9.4 (9.4)	9.2 (9.4)	9.9 (9.3)	7.3 (8.7)	9.9 (9.8)	12.5 (11.6)
**Insurance type**							
Commercial		54.2 (33.1)	58.6 (32.5)	46.8 (32.3)	72.5 (27.6)	48.8 (33.4)	31.5 (29.4)
Medicare		45.8 (33.1)	41.4 (32.5)	53.2 (32.3)	27.5 (27.6)	51.2 (33.4)	68.5 (29.4)
**Urbanicity**							
Rural		6.4 (16.9)	8.5 (21.1)	4.5 (12.2)	4.0 (11.1)	8.1 (17.6)	3.0 (9.2)
Urban		93.5 (16.9)	91.5 (21.1)	95.4 (12.2)	95.9 (11.1)	91.8 (17.6)	96.9 (9.3)
Urbanicity missing		0.1 (1.0)	0.1 (0.9)	0.1 (1.0)	0.1 (1.1)	0.1 (1.3)	0.1 (0.6)

^1^Data presented as the mean (SD) of the proportion in each category for each provider’s patient panel; age is presented as the mean (SD) of the mean age of the patient panel

^2^Only includes patients in the Optum Research Database

^3^Connecticut, Massachusetts, Maine, New Hampshire, Rhode Island, Vermont, New Jersey, New York, Pennsylvania

^4^Illinois, Indiana, Michigan, Ohio, Wisconsin, Iowa, Kansas, Minnesota, Missouri, North Dakota, Nebraska, South Dakota

^5^Washington DC, Delaware, Florida, Georgia, Maryland, North Carolina, South Carolina, Virginia, West Virginia, Alabama, Kentucky, Mississippi, Tennessee, Arkansas, Louisiana, Oklahoma, Texas

^6^Arizona, Colorado, Idaho, Montana, New Mexico, Nevada, Utah, Wyoming, Alaska, California, Hawaii, Oregon, Washington

The mean cumulative incidence of CRC diagnoses was 0.52% among all primary care providers, with rates ranging from a low of 0.28% among OB/GYNs and a high of 0.77% among geriatricians (Table 3). As a measure of preventive care utilization, the incidence rate of patient receipt of influenza vaccination was 51.1% among all primary care providers (Table 3).

**Table 3:** Cumulative incidence rates (mean [standard deviation]) for provider adherence, CRC diagnoses, and influenza vaccination^1^

	Overall Primary (n = 226,534)	Family (n = 88,600)	Internal Medicine (n = 86,164)	OB/Gyn (n = 23,097)	Advanced Practice (n = 28,035)	Geriatrician (n = 638)
Overall adherence^2^	70.0 (16.7)	68.4 (16.6)	69.2 (16.4)	79.5 (14.5)	69.8 (17.5)	63.9 (18.0)
CRC diagnoses^3^	0.5 (1.6)	0.4 (1.2)	0.7 (1.9)	0.3 (1.3)	0.6 (1.9)	0.8 (1.9)
Influenza vaccinations^3^	39.2 (18.5)	42.7 (13.7)	45.0 (13.8)	2.1 (8.5)	40.7 (15.8)	41.1 (12.0)

^1^p < 0.001 for all overall primary provider rates and family versus other provider rates

^2^Assumes 10-year fixed rate

^3^Values based on %

#### Screening adherence

Among patients with a PCP, mean overall adherence with ACS and USPSTF screening guidelines varied by specialty, ranging from a low of 63.9% among geriatricians to a high of 79.5% among OB/GYNs (Table 3). Compared to family medicine providers, internal medicine, OB/GYN, and advanced practice providers all had significantly higher adherence rates.

In the multivariate model, provider factors associated with screening adherence in the enrollee patient sample included provider age, geographic region, and specialty (Supplementary Table 2). Specific characteristics of the enrollee patient panel were also associated with adherence levels, including an increasing adherence rate with higher proportions of high-income patients.

## Discussion

This retrospective, claims-based study examined patient and provider factors associated with CRC screening adherence, as defined by ACS and USPSTF guidelines, for average-risk individuals. Enrollees who had a primary care visit, a preventive care visit, or a main primary care provider had the greatest odds of being screened. Additionally, screened enrollees were more likely to be commercially insured, living in the South, residing in an urban setting, and having at least a high school diploma; however, given the large sample sizes and smaller effect sizes, differences in screening rates by these characteristics may not be clinically meaningful. Female patients appeared more likely to have been screened versus males; however, after adjustment for other demographic and clinical factors, males were more likely to be screened. In Weiss et al., patient-level predictors of screening included increasing age, White race, being married, primarily English-speaking, having commercial insurance, not having congestive heart failure or diabetes, and utilizing more healthcare resources [15]. Consistent with the results from our study, enrollees who had more contact with the healthcare system were more likely to receive preventive care and CRC screening.

Among PCPs, adherence of the patient panel to ACS and USPSTF screening guidelines varied by specialty and ranged from 69.3% among geriatricians to 79.5% among OB/GYNs. In a previous survey study of primary care providers, 77.5% reported using national CRC screening guidelines, but only 51.7% cited recommendations consistent with those guidelines [23]. Additionally, in Weiss et al., CRC screening rates among primary care clinics ranged from 51 to 80%, where, after controlling for multiple patient and clinical factors, an increasing panel size of eligible patients was the only significant predictor of CRC screening [15]. Screening rates reported in our study were also similar to those obtained through patient surveys of US adults aged 50–75 years using both BRFSS data (71.6%) [17] and NHIS data (67.1%) [18].

The lower screening adherence rate among patients of geriatricians in our study was likely due to the medical complexity of those seeing this provider type. Providers must balance the risk of mortality from CRC against that for other comorbidities. A patient with life expectancy estimated at less than 10 years would not likely experience a mortality benefit from CRC screening. [24, 25] Additional factors such as frailty, cognitive function, and patient priorities also play a role in the decision to screen elderly patients [26]. Several studies have documented higher rates of adverse events during and post colonoscopy that increase with age and the presence of comorbid conditions. [27–29] Patients of geriatricians are typically ≥ 65 years of age with complex health care needs and numerous comorbidities that require individualized care beyond that given by a standard family or internal medicine provider.

There are several limitations to this study, including those inherent to claims database analyses. The presence of a diagnosis code on a medical claim does not indicate a positive presence of disease as the diagnosis code may be incorrectly coded or included as rule-out criteria. Information not readily available in the claims data could have affected study outcomes, such as certain clinical and disease parameters. As the claims database included only enrollees with commercial and Medicare Advantage health insurance, the results of this study may not be applicable to the uninsured population or those covered through Medicaid. Uninsured patients face an additional cost barrier and thus, may be less likely to receive screening. The counter-intuitive association between lower education levels and higher rates of CRC screening within this insured population may have been due to confounding with the type of insurance plan available to people in that education category, such as manufacturing jobs with union or contractually ensured levels of health benefits [30]. Information about the plan characteristics were not available for this study. We did not find significant differences in influenza vaccination (a measure of utilization of preventive care measures) by provider type and rates of vaccination were lower than that of CRC screening; however, this may have been an unreliable measure given that influenza vaccinations are frequently received outside of a health plan. Lastly, although advanced practice providers were included as potential providers of primary care, our ability to capture the effect for patients treated by nurse practitioners or physician assistants was limited.

While United States-focused data sources may not translate directly to other countries’ health care systems and populations, similar themes may emerge as additional non-invasive CRC screening methods become available. In a survey-study of European Union countries, screening rates were highest among countries with organized screening programs (from 29.7% in Croatia to 66.7% in the United Kingdom) and those offering both fecal tests and colonoscopy (from 22.7% in Greece to 70.9% in Germany) [31]. A younger age (50–54 years), a longer time since the last physician visit, and a lifestyle score indicating higher CRC risk were associated with lower utilization of screening tests. Patients who reported not having a physician visit within the previous 12 months were 40-60% less likely to have undergone a CRC screening test than patients who had an office visit.

## Conclusions

Among PCPs, adherence of the patient panel to ACS and USPSTF screening guidelines ranged from 69-80% depending on the specialty and provider type. The greatest enrollee-level predictors for CRC screening were having a primary or preventive care visit and having a main primary care provider. These results suggest that increased access to preventive/primary care visits, such as via telemedicine, could improve CRC screening rates. More dependence on home-based screening methods that are non-invasive and non-procedural may reduce some of the burden on patients and providers to complete CRC screening.

## Electronic supplementary material

Below is the link to the electronic supplementary material.

**Additional File 1** Supplementary Table 1. Logistic regression model of the association between female health plan enrollee characteristics and CRC screening status

**Additional File 2** Supplementary Table 2. Ordinary least squares regression model of the association between the characteristics of the provider and their patient panel and adherence with CRC screening guidelines^1^

## Data Availability

The data contained in the Optum Research Database contains proprietary elements owned by Optum and, therefore, cannot be broadly disclosed or made publicly available at this time. The disclosure of this data to third party clients assumes certain data security and privacy protocols are in place and that the third-party client has executed our standard license agreement which includes restrictive covenants governing the use of the data.

## List of abbreviations

ACS: American Cancer Society
CI: Confidence interval
CPT: Current Procedural Terminology
CRC: Colorectal cancer
FIT: Fecal immunochemical test
gFOBT: Guaiac-based fecal occult blood test
HHS: Health and Human Services
ICD-9-CM/ICD-10-CM: International Classification of Diseases 9th and 10th revisions Clinical Modification
mt-sDNA: Multi-targeted stool DNA test
OB/Gyn: Obstetrics/gynecology
OLS: Ordinary least squares
OR: Odds ratio
ORD: Optum Research Database
PCP: Primary care provider
SES: Socioeconomic status
USPSTF: United States Preventive Services Task Force
