# Supplementary material for: Patient and provider factors associated with colorectal cancer screening among average risk health plan enrollees in the US, 2015–2018
Source: BMC Health Serv Res. 2023 May 26;23:550. doi: 10.1186/s12913-023-09474-9 (PMC10223891; doi:10.1186/s12913-023-09474-9)
Supplement: Supplementary file 1 — Additional File 1 Supplementary Table 1. Logistic regression model of the association between female health plan enrollee characteristics and CRC screening status [file 12913_2023_9474_MOESM1_ESM.docx]

Supplementary Table 1. Logistic regression model of the association between female health plan enrollee characteristics and CRC screening status

| **Independent Variables** | **Screened versus Unscreened** | | | | |  |
| --- | --- | --- | --- | --- | --- | --- |
|  | **estimate** | **odds ratio** | **lower 95% CI** | **upper 95% CI** | **p-value** |  |
| **Intercept** | -1.030 | – | – | – | <0.001 |  |
| **Index year** |  |  |  |  | <0.001 |  |
| 2014 | ref. | – | – | – | – |  |
| 2015 | 0.047 | 1.048 | 1.021 | 1.077 | <0.001 |  |
| 2016 | 0.041 | 1.041 | 1.015 | 1.068 | 0.002 |  |
| 2017 | 0.065 | 1.068 | 1.042 | 1.094 | <0.001 |  |
| 2018 | 0.049 | 1.050 | 1.030 | 1.072 | <0.001 |  |
| **Age category** |  |  |  |  | <0.001 |  |
| 50-54 | ref. | – | – | – | – |  |
| 55-59 | -0.107 | 0.898 | 0.883 | 0.914 | <0.001 |  |
| 60-64 | 0.021 | 1.021 | 1.003 | 1.040 | 0.024 |  |
| 65-69 | -0.048 | 0.953 | 0.932 | 0.974 | <0.001 |  |
| 70-74 | -0.084 | 0.920 | 0.899 | 0.941 | <0.001 |  |
| 75+ | -0.144 | 0.866 | 0.842 | 0.891 | <0.001 |  |
| **Region** |  |  |  |  | <0.001 |  |
| Northeast | ref. | – | – | – | – |  |
| Midwest | 0.136 | 1.146 | 1.124 | 1.168 | <0.001 |  |
| South | 0.270 | 1.310 | 1.287 | 1.333 | <0.001 |  |
| West | 0.153 | 1.165 | 1.140 | 1.190 | <0.001 |  |
| Other/missing | -1.842 | 0.159 | 0.116 | 0.216 | <0.001 |  |
| **Race/ethnicity** |  |  |  |  | <0.001 |  |
| White | ref. | – | – | – | – |  |
| African American | 0.060 | 1.062 | 1.042 | 1.082 | <0.001 |  |
| Asian | 0.188 | 1.206 | 1.170 | 1.244 | <0.001 |  |
| Hispanic | 0.231 | 1.260 | 1.236 | 1.285 | <0.001 |  |
| Other | 0.011 | 1.011 | 0.944 | 1.082 | 0.762 |  |
| Race missing/unknown | -0.063 | 0.939 | 0.911 | 0.968 | <0.001 |  |
| No SES information | -0.068 | 0.934 | 0.878 | 0.993 | 0.029 |  |
| **Education** |  |  |  |  | <0.001 |  |
| < 12th grade | 0.289 | 1.336 | 1.223 | 1.459 | <0.001 |  |
| High school diploma | ref. | – | – | – | – |  |
| Some college or Associate  degree | -0.011 | 0.989 | 0.975 | 1.002 | 0.106 |  |
| Bachelor degree/graduate or  professional school degree | 0.058 | 1.060 | 1.038 | 1.082 | <0.001 |  |
| Education missing/unknown/  no SES information | 0.154 | 1.166 | 1.098 | 1.238 | <0.001 |  |
| **Net worth** |  |  |  |  | <0.001 |  |
| < $25,000 | ref. | – | – | – | – |  |
| $25,000- $149,999 | 0.062 | 1.064 | 1.046 | 1.082 | <0.001 |  |
| $150,000- $249,999 | 0.104 | 1.109 | 1.086 | 1.133 | <0.001 |  |
| $250,000- $499,999 | 0.147 | 1.159 | 1.137 | 1.181 | <0.001 |  |
| $500,000- $999,999 | 0.174 | 1.190 | 1.166 | 1.214 | <0.001 |  |
| $1,000,000 + | 0.256 | 1.292 | 1.259 | 1.325 | <0.001 |  |
| Networth missing/unknown/  no SES information | -0.076 | 0.927 | 0.901 | 0.952 | <0.001 |  |
| **Urbanicity** |  |  |  |  |  |  |
| Rural | -0.138 | 0.871 | 0.850 | 0.893 | <0.001 |  |
| Urban | ref. | – | – | – | – |  |
| Urbanicity missing | 0.261 | 1.298 | 0.969 | 1.739 | 0.080 |  |
| **Health plan type** |  |  |  |  | <0.001 |  |
| EPO - Exclusive Provider  Organization | ref. | – | – | – | – |  |
| HMO - Health Plan  Organization | -0.253 | 0.777 | 0.752 | 0.802 | <0.001 |  |
| IND - Indemnity Health Plan | -0.361 | 0.697 | 0.632 | 0.769 | <0.001 |  |
| POS - Point of Service  Health Plan | -0.025 | 0.975 | 0.952 | 0.999 | 0.042 |  |
| PPO - Preferred Provider  Organization | -0.187 | 0.830 | 0.798 | 0.863 | <0.001 |  |
| Multiple of benefit plan/  unknown/missing | -0.280 | 0.756 | 0.728 | 0.785 | <0.001 |  |
| **Indicator for consumer driven health care** |  |  |  |  | <0.001 |  |
| HRA | ref. | – | – | – | – |  |
| HSA | 0.084 | 1.088 | 1.055 | 1.122 | <0.001 |  |
| None - not HRA or HSA | 0.010 | 1.010 | 0.981 | 1.039 | 0.510 |  |
| Indicator for consumer  driven health care missing | 0.049 | 1.050 | 1.008 | 1.094 | 0.020 |  |
| **Baseline Charlson comorbidity index category** |  |  |  |  | <0.001 |  |
| 0 | ref. | – | – | – | – |  |
| 1-2 | 0.046 | 1.047 | 1.033 | 1.062 | <0.001 |  |
| 3-4 | -0.065 | 0.937 | 0.913 | 0.963 | <0.001 |  |
| 5+ | -0.275 | 0.760 | 0.730 | 0.791 | <0.001 |  |
| **Provider type seen** |  |  |  |  |  |  |
| Preventive medicine service | 0.581 | 1.789 | 1.767 | 1.810 | <0.001 |  |
| Family medicine | 0.326 | 1.385 | 1.367 | 1.403 | <0.001 |  |
| Internal medicine | 0.331 | 1.393 | 1.374 | 1.411 | <0.001 |  |
| Obstetrics/gynecology | 0.461 | 1.586 | 1.561 | 1.612 | <0.001 |  |
| Advanced practice | 0.052 | 1.053 | 1.032 | 1.075 | <0.001 |  |
| Geriatrician | 0.020 | 1.020 | 0.851 | 1.223 | 0.826 |  |
| **Number of visits to providers** | 0.007 | 1.007 | 1.005 | 1.009 | <0.001 |  |
| **Baseline AHRQ comorbidities** |  |  |  |  |  |  |
| Thyroid disorders | 0.082 | 1.086 | 1.071 | 1.100 | <0.001 |  |
| Benign neoplasms | 0.206 | 1.228 | 1.208 | 1.249 | <0.001 |  |
| Nutritional deficiencies | 0.088 | 1.092 | 1.076 | 1.108 | <0.001 |  |
| Lipid metabolism disorder | 0.270 | 1.310 | 1.294 | 1.327 | <0.001 |  |
| Other nutritional, endocrine,  and metabolic disorders | 0.080 | 1.084 | 1.069 | 1.098 | <0.001 |  |
| Anemia | 0.072 | 1.074 | 1.054 | 1.096 | <0.001 |  |
| Eye disorders | 0.300 | 1.350 | 1.333 | 1.366 | <0.001 |  |
| Ear conditions | 0.066 | 1.068 | 1.051 | 1.085 | <0.001 |  |
| Diseases of veins and  lymphatics | 0.158 | 1.171 | 1.143 | 1.200 | <0.001 |  |
| Respiratory infections | 0.024 | 1.025 | 1.011 | 1.038 | <0.001 |  |
| Other upper respiratory  disease | 0.048 | 1.049 | 1.032 | 1.067 | <0.001 |  |
| Upper gastrointestinal  disorders | 0.131 | 1.139 | 1.122 | 1.158 | <0.001 |  |
| Gastrointestinal hemorrhage | 0.868 | 2.381 | 2.251 | 2.519 | <0.001 |  |
| Diseases of female genital  organs | 0.162 | 1.176 | 1.159 | 1.192 | <0.001 |  |
| Other inflammatory condition  of skin | 0.078 | 1.081 | 1.058 | 1.104 | <0.001 |  |
| Osteoporosis | 0.213 | 1.238 | 1.212 | 1.264 | <0.001 |  |
| Other connective tissue  disease | 0.058 | 1.060 | 1.047 | 1.073 | <0.001 |  |
| Other bone disease and  musculoskeletal deformities | 0.188 | 1.207 | 1.188 | 1.225 | <0.001 |  |
|  | | | | | | |
