# Supplementary material for: Patient and provider factors associated with colorectal cancer screening among average risk health plan enrollees in the US, 2015–2018
Source: BMC Health Serv Res. 2023 May 26;23:550. doi: 10.1186/s12913-023-09474-9 (PMC10223891; doi:10.1186/s12913-023-09474-9)
Supplement: Supplementary file 2 — Additional File 2 Supplementary Table 2. Ordinary least squares regression model of the association between the characteristics of the provider and their patient panel and adherence with CRC screening guidelines1 [file 12913_2023_9474_MOESM2_ESM.docx]

Supplementary Table 2. Ordinary least squares regression model of the association between the characteristics of the provider and their patient panel and adherence with CRC screening guidelines^1^

| **Independent Variables** | **Pooled cumulative incidence of provider screening adherence** | | | | |
| --- | --- | --- | --- | --- | --- |
|  | **estimate** | **standard error** | **lower 95% CI** | **upper 95% CI** | **p-value** |
| Intercept | 42.385 | 1.179 | 40.075 | 44.695 | <0.001 |
| **Provider Demographics** | | | | | |
| **Age category (as of 2020)** |  |  |  |  |  |
| <50 | ref. | – | – | – | – |
| 50-54 | 0.521 | 0.155 | 0.218 | 0.824 | <0.001 |
| 55-59 | 0.496 | 0.163 | 0.176 | 0.816 | 0.002 |
| 60-64 | 1.077 | 0.165 | 0.753 | 1.400 | <0.001 |
| 65-69 | 0.926 | 0.175 | 0.582 | 1.269 | <0.001 |
| 70-74 | 0.557 | 0.225 | 0.116 | 0.998 | 0.013 |
| 75 | -0.578 | 0.293 | -1.152 | -0.004 | 0.049 |
| missing | 1.438 | 0.142 | 1.159 | 1.717 | <0.001 |
| **Gender** |  |  |  |  |  |
| Female | ref. | – | – | – | – |
| Male | -0.016 | 0.107 | -0.225 | 0.193 | 0.882 |
| Missing | 0.547 | 1.022 | -1.456 | 2.550 | 0.592 |
| **Region** |  |  |  |  |  |
| Northeast | ref. | – | – | – | – |
| Midwest | 2.590 | 0.163 | 2.270 | 2.911 | <0.001 |
| South | 6.627 | 0.150 | 6.334 | 6.921 | <0.001 |
| West^1^ | 3.569 | 0.177 | 3.223 | 3.915 | <0.001 |
| **Race/ethnicity** |  |  |  |  |  |
| White | ref. | – | – | – | – |
| African American | -2.131 | 0.976 | -4.044 | -0.218 | 0.029 |
| Asian | -0.456 | 0.393 | -1.225 | 0.314 | 0.246 |
| Hispanic | 1.496 | 0.541 | 0.435 | 2.557 | 0.006 |
| Other | 0.096 | 0.684 | -1.244 | 1.437 | 0.888 |
| Missing | -0.278 | 0.159 | -0.589 | 0.033 | 0.080 |
| Provider type of practice |  |  |  |  |  |
| Individual | ref. | – | – | – | – |
| Hospital | -2.977 | 2.584 | -8.040 | 2.087 | 0.249 |
| Group practice | -0.599 | 1.014 | -2.587 | 1.388 | 0.555 |
| Other facility | 0.537 | 1.251 | -1.915 | 2.990 | 0.668 |
| Unknown/missing | -2.838 | 1.571 | -5.916 | 0.240 | 0.071 |
| Provider group practice |  |  |  |  |  |
| No group practice | ref. | – | – | – | – |
| Group practice | 0.988 | 0.101 | 0.790 | 1.187 | <0.001 |
| Provider type |  |  |  |  |  |
| Family medicine | ref. | – | – | – | – |
| Internal medicine | -0.238 | 0.110 | -0.453 | -0.023 | 0.030 |
| Obstetrics/gynecology | 3.665 | 0.217 | 3.240 | 4.091 | <0.001 |
| Advanced practice | 2.018 | 0.153 | 1.717 | 2.318 | <0.001 |
| Geriatrician | -2.697 | 0.854 | -4.370 | -1.023 | 0.002 |
| **Characteristics of the Provider's Patient Panel** | | | | | |
| **Age category (%)** |  |  |  |  |  |
| 50-54 | ref. | – | – | – | – |
| 55-59 | -0.040 | 0.007 | -0.053 | -0.026 | <0.001 |
| 60-64 | -0.078 | 0.007 | -0.091 | -0.065 | <0.001 |
| 65-69 | -0.086 | 0.007 | -0.100 | -0.071 | <0.001 |
| 70-74 | -0.141 | 0.007 | -0.155 | -0.126 | <0.001 |
| 75 | -0.353 | 0.008 | -0.370 | -0.337 | <0.001 |
| **Gender (%)** |  |  |  |  |  |
| Female | ref. | – | – | – | – |
| Male | -0.145 | 0.004 | -0.152 | -0.138 | <0.001 |
| **Race/ethnicity** |  |  |  |  |  |
| White | ref. | – | – | – | – |
| African American | 0.102 | 0.004 | 0.095 | 0.109 | <0.001 |
| Asian | 0.030 | 0.007 | 0.017 | 0.043 | <0.001 |
| Hispanic | 0.088 | 0.004 | 0.081 | 0.096 | <0.001 |
| Other or Unknown | 0.019 | 0.022 | -0.024 | 0.061 | 0.390 |
| Missing | 0.064 | 0.013 | 0.039 | 0.089 | <0.001 |
| **Education (%)** |  |  |  |  |  |
| <12th grade | 0.186 | 0.022 | 0.143 | 0.229 | <0.001 |
| High school diploma | ref. | – | – | – | – |
| Some college or Associate  degree | -0.022 | 0.003 | -0.028 | -0.016 | <0.001 |
| Bachelor degree/graduate or  professional school degree | -0.013 | 0.005 | -0.022 | -0.003 | 0.008 |
| Education missing or no  SES information | 0.081 | 0.015 | 0.052 | 0.111 | <0.001 |
| **Net worth (%)** |  |  |  |  |  |
| < $25,000 | ref. | – | – | – | – |
| $25,000- $149,999 | 0.068 | 0.006 | 0.056 | 0.080 | <0.001 |
| $150,000- $249,999 | 0.104 | 0.008 | 0.089 | 0.120 | <0.001 |
| $250,000- $499,999 | 0.158 | 0.006 | 0.146 | 0.171 | <0.001 |
| $500,000- $999,999 | 0.166 | 0.006 | 0.154 | 0.179 | <0.001 |
| $1,000,000 + | 0.243 | 0.007 | 0.229 | 0.257 | <0.001 |
| Net worth missing or no SES  information | 0.036 | 0.009 | 0.018 | 0.053 | <0.001 |
| **Urbanicity (%)** |  |  |  |  |  |
| Rural | -0.026 | 0.003 | -0.032 | -0.021 | <0.001 |
| Urban | ref. | – | – | – | – |
| Urbanicity missing | 0.047 | 0.044 | -0.039 | 0.134 | 0.282 |
| **Health plan type (%)** |  |  |  |  |  |
| EPO - Exclusive Provider  Organization | 0.064 | 0.009 | 0.047 | 0.081 | <0.001 |
| HMO - Health Plan  Organization | ref. | – | – | – | – |
| IND - Indemnity Health Plan | -0.014 | 0.021 | -0.056 | 0.027 | 0.501 |
| POS - Point of Service  Health Plan | 0.081 | 0.006 | 0.068 | 0.093 | <0.001 |
| PPO - Preferred Provider  Organization | 0.192 | 0.004 | 0.184 | 0.200 | <0.001 |
| Multiple of benefit plan | -0.052 | 0.154 | -0.354 | 0.250 | 0.737 |
| Health plan type missing | 0.193 | 0.003 | 0.186 | 0.199 | <0.001 |
| **Indicator for consumer driven health care (%)** |  |  |  |  |  |
| HRA | ref. | – | – | – | – |
| HSA | 0.097 | 0.010 | 0.078 | 0.116 | <0.001 |
| None - Not HRA or HSA | 0.004 | 0.009 | -0.013 | 0.021 | 0.631 |
| Indicator for consumer  driven health care missing | 0.000 | 0.011 | -0.021 | 0.021 | 0.984 |
| **Number of patients for each provider (%)** | 0.004 | 0.000 | 0.004 | 0.005 | <0.001 |
| **CRC diagnoses (%)** | -0.152 | 0.029 | -0.208 | -0.096 | <0.001 |
| **Influenza vaccinations (%)** | 0.173 | 0.003 | 0.166 | 0.179 | <0.001 |
| ^1^Wolf AMD, Fontham ETH, Church TR, et al. Colorectal cancer screening for average-risk adults: 2018 guideline update from the American Cancer Society. CA Cancer J Clin 2018;68(4):250-281. | | | | | |
